# Editorial: The role of self-perception in mental health: current insights on self-esteem and self-schemas

**DOI:** 10.3389/fpsyt.2026.1852676

**Published:** 2026-05-19

**Authors:** Manel Monsonet, Tecelli Domínguez-Martínez, Regina Espinosa

**Affiliations:** 1 Departament de Psicologia Clínica i de la Salut, Universitat Autònoma de Barcelona, Barcelona, Spain; 2 Department of Psychology, University of Illinois at Urbana-Champaign, Urbana-Champaign, IL, United States; 3 Centro de Investigación en Salud Mental Global, Dirección de Investigaciones Epidemiológicas y Psicosociales, Instituto Nacional de Psiquiatría Ramón de la Fuente Muñiz, Mexico City, Mexico; 4 Faculty of Health Sciences - HM Hospitals, Camilo Jose Cela University, Madrid, Spain; 5 HM Hospitals Health Research Institute, Madrid, Spain

**Keywords:** mental health, psychopathology, self-esteem, self-perception, self-schemas, self-views

The way we perceive ourselves has fundamental implications in almost all aspects of life such as psychological well-being, social functioning, and physical and mental health. Self-perception is the process through which individuals observe, interpret, and form judgments about their own traits, behaviors, and internal states—including emotions, attitudes, and motivations. This dynamic process serves as the fundamental scaffolding upon which individuals construct their self-concept, briefly defined as a mental representation of one’s own person (1). Self-concept can be understood as the cognitive and affective representation of the self as an object of knowledge that encompasses an individual’s relatively stable beliefs, attitudes, and feelings about their personal attributes (2). However, self-concept is a broad, overarching construct that encompasses multiple, distinct dimensions. Among these dimensions, self-esteem and self-schemas stand out as particularly influential for mental health, functioning as both a risk and a protective factor in the development of psychopathology.

Self-esteem is defined as the evaluative component of self-concept, entailing a relatively stable favorable or unfavorable attitude towards the self (3). Impaired self-esteem has historically been implicated in psychopathology and constitutes a key element in several etiological theories of mental disorders. In fact, low self-esteem is a diagnostic criterion for depressive disorders and is often present as an associated feature in many other conditions. Moreover, elevated self-esteem is also observed in other mental disorders such as bipolar disorders and narcissistic personality disorders.

Self-schemas are relatively stable cognitive structures composed of generalizations about the self (e.g., “I am smart” or “I am a failure”) that play a critical role in the appraisal of experience (4). Unlike self-esteem—which entails a global evaluation of personal worth—self-schemas function as cognitive templates that guide the processing and interpretation of self-relevant information. Self-schemas have been implicated in psychopathology, especially when negative in content, and have become central to cognitive models of various disorders, including anxiety, mood, psychotic, and personality disorders.

Research indicates that self-views are linked to various psychiatric symptoms and disorders, with impairments in self-esteem and self-schemas potentially preceding the onset of full symptoms or arising as a consequence of a psychiatric disorder (4–7). Therefore, this Research Topic aims to gather new evidence on the relationship between self-esteem, self-schemas, and psychiatric symptoms and disorders across clinical settings and nonclinical populations. A central aim is to understand how damaged self-concepts influence the onset and trajectory of psychiatric conditions, as well as how psychiatric symptoms subsequently affect self-view.

Studies on this Research Topic provide insights into some of these questions. Two studies have focused specifically on the relationship between self-schemas, self-esteem, and psychological distress. In a post-conflict context in Mosul, Iraq, Ibrahim et al. identify self-esteem and positive self-schemas as protective factors against psychological distress, whereas negative self-schemas emerge as a risk factor. Monsonet et al. explore the interplay between self-schemas and affective temperament in a large sample of young adults. Their work provides evidence that both positive and negative self-schemas function as key mediators between distinct affective temperaments and the expression of anxious and depressive symptoms.

Using ecological momentary assessment, Gütges et al. investigated the stability of self-appraisal and reflected appraisal in relation to daily mood changes. Self-appraisal (internal self-evaluation) and reflected appraisal (perceived evaluation by others) are central to understanding the stability of self-perception. Their findings indicate that variability in both self- and reflected appraisal was positively related to mood changes, with a stronger relationship observed for self-appraisal.

Two studies have explored the impact of self-esteem and self-schemas on paranoid beliefs, both using classical conditioning paradigms to modify implicit self-esteem. One study found that implicit self-evaluations are malleable and may reduce threatening interpretations of social information, alongside preliminary neurophysiological changes in regions linked to emotional reactivity and mentalization (Trucharte et al.). In contrast, another study using a similar conditioning approach to manipulate self- and other-schemas did not find significant effects on paranoia or self-esteem, although changes were observed in attachment-related states (Martínez et al.). Together, these findings suggest that while implicit self-related processes can be experimentally modified, their impact on paranoid beliefs may depend on how these processes are targeted and measured.

Extending this research beyond paranoia, a third study examined appearance-related self-perception. Individuals with jaw deformities showed higher levels of body dysmorphic traits and a marked attentional bias toward appearance-relevant facial features, which was associated with increased appearance-related concern (Matsuzawa et al.). These findings indicate that disturbances in self-perception may also manifest through attentional mechanisms, extending current conceptualizations of self-schemas beyond evaluative beliefs about the self.

Two qualitative studies have involved patients with bipolar disorders (BD). Ponticello et al. explored how people with BD perceive their condition, including the relationship between BD and the self. Unlike previous studies suggesting that “BD gradually becomes part of the self”, participants acceptance of their condition allowed them to separate BD from their sense of self while integrating it into their way of being and living. Oblak et al. presented an interdisciplinary case study of a patient with comorbid BD and related difficulties in social cognition and affectivity. Through a multidisciplinary analysis of qualitative materials, the authors discuss how the patient’s lived experience and self-narrative offer a novel framework for understanding his psychopathology. Finally, Tan et al. advanced our understanding of cognitive biases in depression by examining the predictive accuracy of depressive symptoms using various measures derived from a widely used self-schema task (the SelfReferential Encoding Task; SRET) across clinical, community, and healthy samples.

In sum, this Research Topic underscores self-perception as a fundamental mechanism in mental health. Across diverse methodologies and populations — from post -conflict settings to intensive longitudinal assessments— self-perception emerges not merely as a passive agent of psychopathology but as an active and malleable process capable of shaping its development and course. Clinically, these findings suggest that interventions targeting self-perception may hold transdiagnostic potential.
